# Development of a Serum Metabolome‐Based Test for Early‐Stage Detection of Multiple Cancers

**DOI:** 10.1002/cnr2.70042

**Published:** 2024-11-19

**Authors:** Rajnish Nagarkar, Mamillapalli Gopichand, Suparna Kanti Pal, Ankur Gupta, Najmuddin Md Saquib, Ahmad Ahmad, Ganga Sagar, Kanury V. S. Rao, Zaved Siddiqui, Imliwati Longkumer

**Affiliations:** ^1^ HCG Manavta Cancer Centre Nashik Maharashtra India; ^2^ HealthCare Global Enterprises Limited, City Cancer Centre Vijayawada India; ^3^ Indoriv Clinical Kolkata India; ^4^ Department of Radiotherapy IPGME&R Kolkata India; ^5^ PredOmix Technologies Private Limited Gurugram India; ^6^ PredOmix Health Sciences Private Limited Singapore Singapore; ^7^ North East Cancer Hospital and Research Institute Jorabat, Guwahati Assam India

**Keywords:** artificial intelligence, cancer, metabolomics

## Abstract

**Background:**

Detection of cancer at the early stage currently offers the only viable strategy for reducing disease‐related morbidity and mortality. Various approaches for multi‐cancer early detection are being explored, which largely rely on capturing signals from circulating analytes shed by tumors into the blood. The fact that biomarker concentrations are limiting in the early stages of cancer, however, compromises the accuracy of these tests. We, therefore, adopted an alternate approach that involved interrogation of the serum metabolome with machine learning‐based data analytics. Here, we monitored for modulations in metabolite patterns that correlated with the presence or absence of cancer. Results obtained confirmed the efficacy of this approach by demonstrating that it could detect a total of 15 cancers in women with an average accuracy of about 99%.

**Aims:**

To further increase the scope of our test, we conducted an investigator‐initiated clinical trial involving a total of 6445 study participants, which included both cancer patients and non‐cancer volunteers. Our goal here was to maximize the number of cancers that could be detected, while also covering cancers in both females and males.

**Methods and Results:**

Metabolites extracted from individual serum samples were profiled by ultra‐performance liquid chromatography coupled to a high‐resolution mass spectrometer using an untargeted protocol. After processing, the data were analyzed by our cancer detection machine‐learning algorithm to differentiate cancer from non‐cancer samples. Results revealed that our test platform could indeed detect a total of 30 cancers, covering both females and males, with an average accuracy of ~98%. Importantly, the high detection accuracy remained invariant across all four stages of the cancers.

**Conclusion:**

Thus, our approach of integrating untargeted metabolomics with machine learning‐powered data analytics offers a powerful strategy for early‐stage multi‐cancer detection with high accuracy.

**Trial Registration:** Registration No: CTRI/2023/03/050316

## 1 Introduction

Cancer threatens to emerge as the leading cause of premature mortality across the globe [1, 2]. Current therapies at best provide only a marginal prolongation of survival and more effective therapies are clearly needed [3]. On the other hand, population‐scale detection of cancers in their early stage of development and/or progression offers a feasible strategy for reducing cancer‐associated mortality [4, 5, 6, 7, 8, 9]. Besides impacting on mortality, early detection also helps to reduce treatment cost and improve quality of life by minimizing disease‐ and treatment‐related morbidity [10, 11]. Unfortunately, though, clinically accepted screening tests are currently available for only five cancers (breast, cervical, colorectal, lung, and prostate) [12, 13, 14, 15, 16]. While these tests have undoubtedly contributed to reducing cancer‐specific mortality their benefits have, nonetheless, been mitigated by disparities in adherence and a high false‐positivity rate that results in overdiagnosis and over‐treatment [17, 18, 19, 20, 21, 22, 23, 24]. Thus, while on the one hand there is an urgent need to develop more effective approaches for detecting a broader spectrum of cancers, the need for improving the sensitivity and specificity of such detection methods is also an imperative for mitigating the problem of over diagnosis [25, 26].

Multi‐cancer early‐detection (MCED) is a relatively new approach that is now gaining traction as a promising strategy for detecting multiple cancers in their early stages [27]. To date, methods based on this approach have relied on blood‐based detection of either circulating tumor cells (CTCs) [28], or circulating tumor DNA (ctDNA), or on other such circulating analytes shed by tumors into blood [29, 30, 31, 32, 33]. Despite the promise shown by such MCED tests, however, their utility appears to be currently hampered by some seemingly intractable limitations. Prominent among these is the low circulating concentrations of biomarkers, especially in early‐stage cancers, which limits the detection sensitivity that can be achieved [34]. Furthermore, in addition to tumor burden, other patient‐related clinical factors have also been found to influence ctDNA detection, thereby complicating interpretation of the results [35]. Thus, more work is clearly needed to optimize the performance of such tests [36].

In contrast to the MCED‐targeted approaches currently being employed, we had adopted an alternate strategy that exploited the serum metabolome towards identification of metabolite patterns that correlated with the presence or absence of cancer. The rationale for our approach was founded on the fact that, given the significant role that they play in biological regulation—integrating the effects of both genetics and lifestyle factors on health—the metabolite composition provides an accurate descriptor of the health of an individual [37, 38]. This aspect seemed, to us, to be particularly relevant for cancer given that metabolic reprogramming constitutes an important hallmark of cancer cells [39, 40]. Consequently, we developed a methodology wherein untargeted serum metabolome profiling by mass spectrometry was integrated with machine learning (ML)‐driven data analysis to extract cancer‐specific metabolite signatures. We first confirmed the feasibility of this approach by demonstrating its ability to simultaneously detect early‐stages (Stage‐0/I) of the four women‐specific cancers (breast, endometrial, cervical, and ovarian) with an accuracy of around 98% [41]. Importantly, in a subsequent study we showed that the accuracy of this test was retained in a single‐blinded clinical study where serum samples were obtained from subjects in the field [42]. These combined results suggested to us that our method indeed had potential for further development as a cancer screening test [41, 42].

In addition to detection accuracy, a useful MCED test should be able to detect as many cancer types as possible to enhance cancer detection rate in the target population. Therefore, we followed up on our earlier studies [41, 42] by exploring whether our method could be adopted to detect additional cancer types beyond the four women‐specific cancers tested. Here again our emphasis was on detection of early stage (i.e., Stage‐I) cancers. As shown in our recent report, we could indeed expand the scope of our test to detect a total of 15 cancers in women with an average sensitivity of > 99% and a specificity of 99.3% [43]. It is worthwhile noting here that such a high detection accuracy for Stage‐I cancers has not been achieved by any of the existing screening tests, including other MCED tests, so far [27, 28, 29, 30, 31, 32, 33]. In the present study we took our work a step further by conducting an investigator‐initiated clinical trial wherein we obtained serum samples from a total of 6445 study participants, roughly divided equally between females and males. Of these, 2831 subjects were cancer patients who collectively presented a total of 30 different cancer types. The remaining 3614 serum samples were from non‐cancer volunteers. Results obtained here demonstrate that our test platform could detect all 30 cancers, in both males and females, with high accuracy. An average detection sensitivity of 97.5% was obtained at a specificity of 99.2%. Significantly, the high detection accuracy was maintained across all the four stages of cancer, as well as in all the adult age groups tested.

## 2 Materials and Methods

### 2.1 Study Design and Participant Sampling

Serum samples for the present study were obtained from through a case‐control, single arm, observational study in the Indian population, where samples were collected from a total of 6445 participants. Sample collection was achieved within the period spanning from April to December 2023. Of these, 2831 were participants who had been newly diagnosed with cancer but were as yet treatment naïve. Cancer diagnosis in this group included confirmation through a histological examination of biopsies (see Table S1). The remaining 3614 participants were non‐cancer volunteers (normal controls). This latter group of volunteers were selected on the basis of the following criteria: (i) no known history of any type of cancer; (ii) no suspicion of cancer based on investigation reports—such as hematology, biochemistry, metabolic panel, urinalysis, and so forth—generated within 30 days of blood sampling; (iii) physical examination, including vital sign assessment, to ensure that the subject does not have any underlying signs of undetected cancerous growth or symptoms. This latter group was considered to represent the normal control group for our study. This study was conducted as an investigator‐initiated study (Registration No. CTRI/2023/03/050316) and sample collection was initiated only after approval from the Institutional Ethics Committees of the respective centers. Informed consent was obtained from all study participants and the study was conducted in accordance with the Good Clinical Practice Guidelines as laid out by the International Conference on Harmonization. Up to 3 mL of blood was collected from the individual participants, from which serum was subsequently prepared. Samples were then anonymized before submitting to the analytical team (A.G., G.S., Z.S., K.V.S.R., N.M.S.) for analysis. A unique identity number (identifier) was assigned to each sample, which was used for queries related to sample handling, task details, and results. Samples were stored at −80°C until required (Table 1).

**TABLE 1:** Demography and clinical profile of study participants.

Cancer (*n* = 2831)		Non‐cancer (*n* = 3614)	Total (*N* = 6445)
Age group, *n* (%)			
20–30 years	147 (5.2)	683 (18.85)	830 (12.9)
31–40 years	381 (13.5)	685 (18.96)	1066 (16.5)
41–50 years	711 (25.1)	734 (20.32)	1445 (22.4)
51–60 years	805 (28.4)	740 (20.49)	1545 (24.0)
61–70 years	558 (19.7)	556 (15.39)	1114 (17.3)
> 70 years	229 (8.1)	216 (5.98)	445 (6.9)
Gender, *n* (%)			
Female	1341 (47.4)	1739 (48.1)	3080 (47.8)
Male	1490 (52.6)	1875 (51.9)	3365 (52.2)
Body mass index (kg/m^2^), *n* (%)			
< 30	2760 (96.4)	3144 (87.01)	5904 (87.01)
> 30	71 (3.6)	470 (12.98)	541 (12.98)
Clinical cancer stage, *n* (%)			
I	481 (17.0)		
II	1136 (40.1)		
III	898 ((31.7)		
IV	310 (11.0)		
NS^a^	6 (0.2)		

^a^ NS: Cancers of unknown primary origin, which are not staged.

### 2.2 Metabolite Extraction and UPLC‐MS/MS Analysis

Metabolites were separately extracted from 10 μL of each serum sample, processed, and analyzed using an untargeted approach by ultra‐pressure liquid chromatography coupled to mass spectrometry (UPLC‐MS/MS) as previously described [41, 43]. Here, samples were randomized prior to analysis so as to minimize generation of any class‐specific biases in the metabolome profiles. Pooled positive (PC) and negative control (NC) samples were also included to monitor quality of the LC‐MS/MS run as previously described ([41], Figure S2). The mass spectrometry data thus generated was first subjected to mass error correction using the modified virtual lock mass (VLM) and combining it with metabolite annotation using the Human Metabolome Database (HMDB) as previously described [41, 43]. This exercise resulted in an initial matrix of 8312 metabolites or features, which was subsequently processed further as described below.

### 2.3 Development of the Cancer Detection AI Algorithm

**FIGURE 1:**
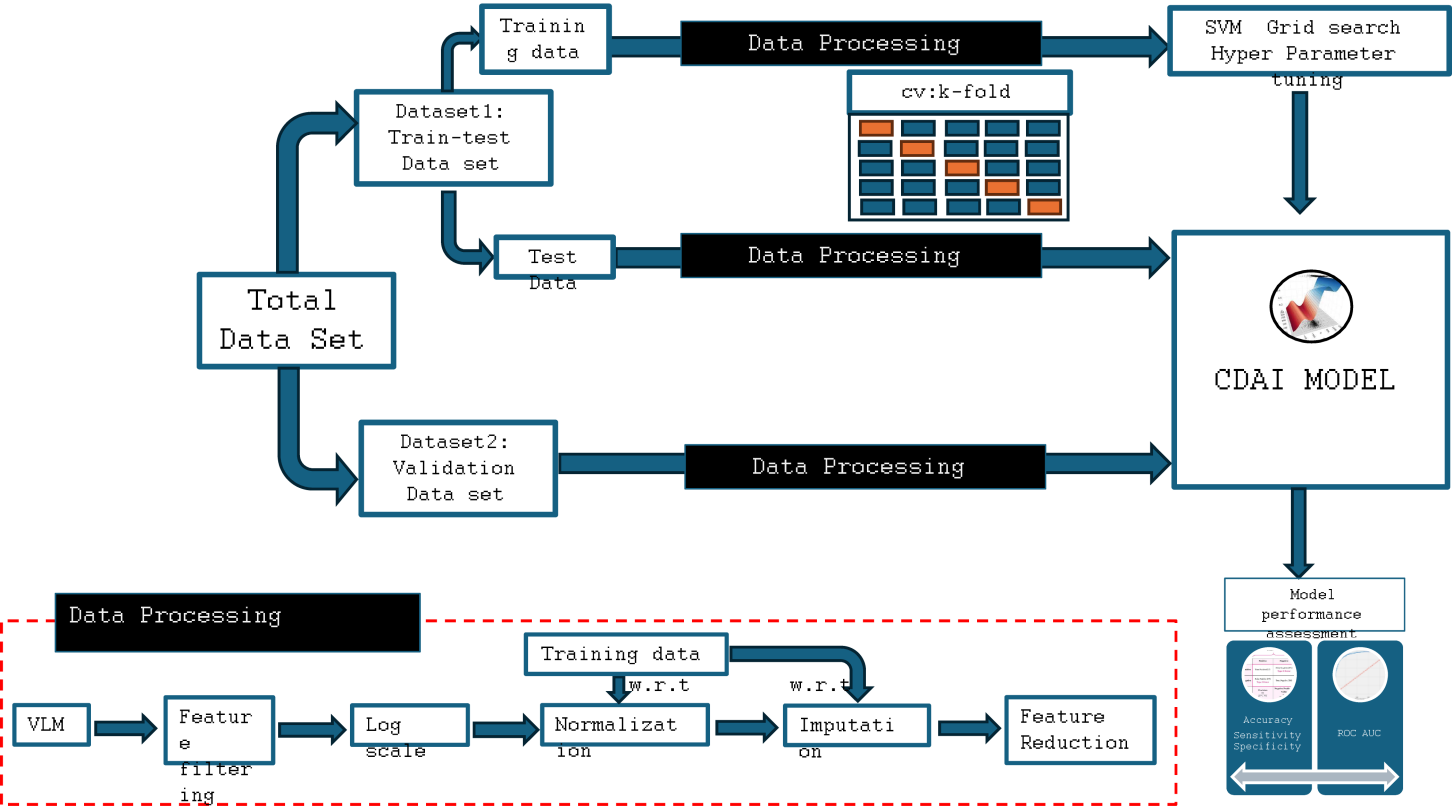
Pipeline for data processing and development of the CDAI algorithm. Figure illustrates the workflow for development of the CDAI model, which also includes the data pre‐processing steps employed. A detailed explanation of the individual steps is provided in the Section 2.

For development of an AI model that could accurately differentiate all the 30 cancers listed in Table 2 from normal controls, we recognized the likelihood that the complexity of technical and biological processing of MS based data may produce inherent systemic biases. These non‐biological signals affect the true biological signal and can be detrimental for downstream analysis. Consequently, before implementing the AI workflow, we included a series of pre‐processing procedures in an attempt to manage this variance. While Figure 1 provides a schematic of the overall procedure, the specifics of each step are as follows.

- *Feature matrix generation* (*inclusion of mass error*):

**TABLE 2:** Class‐specific and stage‐specific distribution of the cancer samples.

No.	Cancer type	Female (*n*)	Male (*n*)	Total (*N*)	Clinical Stage (*n*)			
					I	II	III	IV
1	Breast	262	—	262	36	133	77	16
2	Endometrial	70	—	70	20	26	19	5
3	Cervical	137	—	137	27	58	40	12
4	Ovarian	155	—	155	28	64	48	15
5	Lung	68	158	226	40	55	80	51
6	AML	36	50	86	10	15	29	32
7	Thyroid	58	41	99	16	46	32	5
8	Melanoma	18	47	65	24	26	14	1
9	Colorectal	82	127	209	20	81	90	18
10	Kidney	16	39	55	8	25	17	6
11	NHL	19	28	47	13	15	9	10
12	Pancreatic	19	40	59	9	25	17	8
13	Liver and bile	35	78	113	36	33	32	12
14	Gastric	62	132	194	38	90	57	9
15	Head and neck	154	397	551	80	262	147	61
16	Esophageal	67	76	143	26	61	42	14
17	Bladder	10	22	32	4	12	14	2
18	Brain and CNS	8	34	42	11	13	14	4
19	Multiple myeloma	3	15	18	0	7	7	4
20	Gall bladder	18	17	35	3	18	13	1
21	Sarcoma	22	23	45	6	10	23	6
22	Prostate	—	122	122	17	37	55	13
23	Testicular	—	4	4	0	2	2	0
24	Vulvar	5	—	5	0	1	2	2
25	Anal	6	3	9	2	3	3	1
26	Vaginal	2	—	2	0	0	2	0
27	Penile	—	18	18	4	7	7	0
28	Unknown primary origin	4	2	6	—	—	—	—
29	Germ cell tumor	2	12	14	0	10	3	1
30	Squamous cell carcinoma	4	4	8	3	1	3	1

Abbreviations: AML, acute myeloid Leukemia; NHL, non‐Hodgkin's lymphoma.

Mass Errors are common in metabolomics data, even with very strict protocols in place to prevent fluctuation in data. This results in a shift in the mass (*m/z*) of metabolite in different runs/ samples. Random mass variance can serve as a confounding factor for downstream AI model development as the latter needs common features to perform any pattern recognition. Here in this study, we have followed adaptive VLM mass alignment technique as described earlier [41]. This generated an initial feature set of 8312 known serum metabolites.

ii *Data filtering* (*DF*):

The feature matrix generated in the previous section were filtered using the various metabolomics databases, as well as manual curation, to remove plant‐derived, drug derived, and other metabolites whose human origin could not be unambiguously ascertained. This step reduced the feature set to 5104 metabolites.

iii *Data Scaling and normalization*:

All LC‐MS based intensities were first log scaled with base 10. The samples were run across various batches having different cancer signatures and produced an abundance of technical variation. To counter this variability the log‐scaled data was normalized using a quantile‐based approach. The normalization technique was modified to align with our single sample‐based testing procedures. For this the train dataset was used as reference for quantile normalization in any testing or validation sample.

iv *Handling missing values*:

Missingness in MS‐based Metabolomics data occurs generally where a metabolite gets unquantified due to technical or biological reasons. To handle this shortcoming, we added a missing value handling step, where we used Multiple Imputation by Chained Equations (mice) forest algorithm for imputation. Mice forest iteratively predicts the missing value for the feature using a series of predictive models. We have used LightGBM regressor for this purpose. The steps for the MICE algorithm are as follows:

Step 1: For a continuous dataset such as ours, an initial guess is set for each feature using the mean respective feature mean across the dataset.

Step 2: For predictive modeling and assessment of the LightGBM model, the dataset is split into two sections: the training data, and the prediction data. Following step 1 After feeding the regressor with these training and prediction sets, the predicted data is imputed at the proper locations. One iteration is completed after all the values have been imputed.

Step 3: Up until a halting condition is met, the previous step is repeated. In successive rounds, the iterative process confirms that the algorithm is set to work on an improved dataset. Until a certain iteration limit is reached or the sum of squared discrepancies between the current and prior imputation grows, the procedure is repeated. We used five iterations for our dataset.

This generated a fitted imputation model from the training dataset which was used to missingness handling in testing or validation samples.

v *Feature reduction*:

Data adaptive feature filtering techniques are crucial in high dimensional dataset for robust training–testing validation exercises by reducing the chances of data overfitting. Here in our study, we removed the lowest *k* (*k* = 20) percent of features based on the size of the dataset on abundance across samples. This step reduced the feature matrix to 2709 metabolic features.

vi *ML model development*:

Sample based analysis is cardinal for the model to have a potential application in clinical settings. All of the above steps were tailored for that in our analysis. Here, to curb batch effect in the mass spectrometric dataset we used a series of the more commonly used post‐acquisition sample normalization methods involving log‐scaling, quantile normalization and mice forest based imputation. Figure S3 depicts the batch effect correction that could be achieved using the data processing pipeline. The pre‐processed data was then fed into the ML models for the cancer versus normal control classification objective. The dataset defined in Table 2 was first split in the training–testing set and the validation set. The distribution of the cancer and the normal controls in training‐test and validations sets is given in the Tables S2 and S3. Pooling the cancer subtypes across 30 cancers we used 1445 samples in the training–testing set. Along with this inclusion of 1812 samples from cancer free normal controls yielded a total of 3257 samples for the training–testing step (Table S2). The comprehensive flow chart of the process is shown in Figure 1. Owing to non‐linearity in the data between classification variables and feature variation, and the limitation of samples, we used nonlinear Support vector machine (SVM) for the purpose. This method's main goal is to use various kinds of kernel functions to project nonlinear separable samples onto a higher dimensional space. The goal of SVM‐based classification is to use kernel functions to maximize the margin between the support vectors. This kernel is constructed by computing the pair wise distance in the training dataset. The Radial basis function or RBF (Gaussian) kernel function is one of the most widely used kernel methods in high dimensional biological classification problems. This Gaussian kernel and the RBF function are given below;

RBF (SVM)

$$F\left(x\right)=\sum_{i}^{N}\alpha_{i}\frac{y_{i}\exp\left(-{\left\|x-x_{i}\right\|}^{2}\right)}{2\sigma^{2}}+b$$

Gaussian kernel

$$k\left(x,x^{\prime }\right)=\exp\frac{\left(-{\left\|x-x^{\prime }\right\|}^{2}\right)}{2\sigma^{2}}$$

where alpha is the weight, *k* is the Gaussian kernel, sigma is, *N* is the number of training sample, $x_{i}^{\prime }s$ are the support vectors, sigma is, as usually defined in a Gaussian distribution, is standard deviation, *b* is the intercept.

**FIGURE 2:**
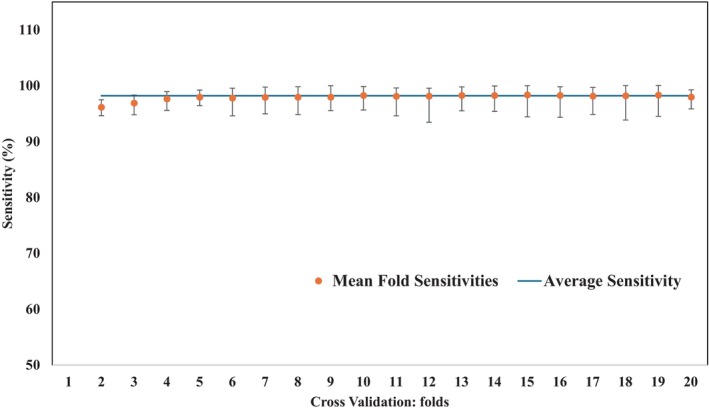
*k*‐fold cross validation of the CDAI algorithm. To cross‐validate the model we used the *k* (*k* = 20) fold cross validation where the train test dataset was split into 20 parts. Figure shows the results for test sensitivities for each fold along with their 95% CI. The blue line shows the average sensitivity of the CDAI model.

We used the Grid search method to approximate the optimal value for the hyperparameters namely *σ* (standard deviation of Gaussian kernel), C (regularization hyperparameter) and so forth. The model was assessed using sensitivity, specificity, accuracy, and AUC‐ROC scores. This was followed by a *k*‐fold cross validation step where we used a 20‐fold approach. The training–testing dataset was first split into 20 equal folds and each fold was tested one by one. Cross validation score for each fold was evaluated along with the 95% CI. The results for the cross‐validation of the model is shown in Figure 2, where the average sensitivity, specificity and accuracy of the model were defined. This generated the cancer detection artificial intelligence (CDAI) model which stands for CDAI model. The CDAI model was able to distinguish cancer and normal control samples with high sensitivity, specificity when a cut‐off of zero was applied on the scores obtained from SVM RBF. The formulae used for the sensitivity, specificity, and accuracy used are given below.

**Table:** 

	Predicted		
Actual		Negative	Positive
	Negative	True negative (TN)	False positive (FP)
	Positive	False negative (FN)	True positive (TP)

$$\text{Accuracy}:\frac{\mathrm{TP}+\mathrm{TN}}{\mathrm{TP}+\mathrm{TN}+\mathrm{FP}+\mathrm{FN}}$$

$$\text{Sensitivity}:\frac{\mathrm{TP}}{\mathrm{TP}+\mathrm{FN}}$$

$$\text{Specificity}:\frac{\mathrm{TN}}{\mathrm{TN}+\mathrm{FP}}$$

vii *Validation*

**FIGURE 3:**
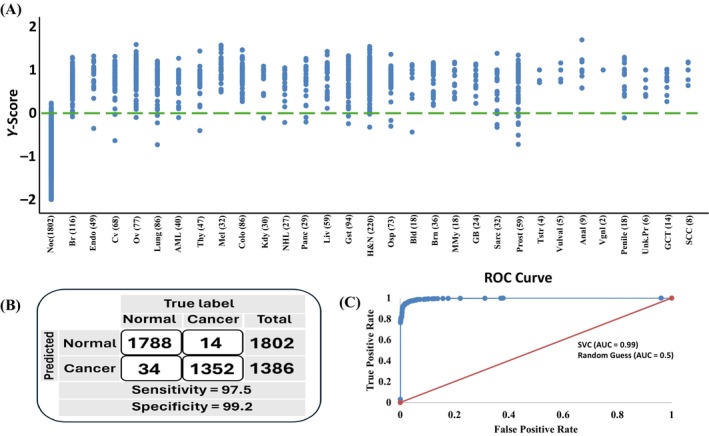
Differentiating cancer samples from normal controls using the CDAI algorithm. (A) This panel shows the results obtained for normal samples, and the samples from each of the cancer types tested in the validation set, using the CDAI algorithm. A scatter plot depicting the distribution of *y*‐scores for individual samples either in the normal control group, or in each of the 30 cancer types tested, is presented here. Whereas the normal control group is denoted as Noc, individual cancer types are abbreviated as Br, breast cancer; Endo, endometrial cancer; Cv, cervical cancer; Ov, ovarian cancer; Lung, lung cancer; AML, acute myeloid leukemia; Thy, thyroid cancer; Mel, melanoma, Colo, colorectal cancer; Kdy, kidney cancer; NHL, Non‐Hodgkin's lymphoma; Panc, pancreatic cancer; Liv, liver and bile duct cancer; Gst, gastric cancer, H&N, head and neck cancer; Osp, oesophageal cancer; Bld, bladder cancer; Brn, brain and CNS cancer; MMY, multiple myeloma; GB, gall bladder cancer; Sarc, sarcoma; Prost, prostate cancer; Tstr, testicular cancer; Vulval; vulval cancer; Anal, anal cancer; Vgnl, vaginal cancer; Penile, penile cancer; Unk. Pr, cancer of unknown primary origin; GCT, germ cell tumors; SCC, squamous cell carcinoma. Panel (B) gives the resultant confusion matrix obtained from this data, after taking a *y*‐score of 0 as the threshold score for distinguishing cancer‐positive samples from normal controls. Values obtained for sensitivity and specificity are also given. The ROC‐AUC probability curve plot, as an indicator of CDAI performance, is shown in panel (C). The area under the curve (AUC) obtained was 0.99.

High dimensional ML models are vulnerable to overfitting during training–testing procedures. However, we have employed strict procedures to ensure that the model would likely get validated in independent dataset. For this we used the independent dataset which consisted of 1386 cancer samples, distributed across the 30 cancers as well as the four cancer stages, in addition to 1802 normal control samples. The sample distribution of the validation set is given in Table S3. Following the data processing pipeline the validation set was fed into the CDAI model and scores were generated. The *y*‐score plot shown for the validation set is shown in Figure 3 clearly displays the model's ability to distinguish the cancer and normal samples. The confusion matrix and the AUC ROC curve for the validation set were also generated using the CDAI model.

## 3 Results

### 3.1 Study Design and Details of Participants

Serum samples were obtained through a case‐control, observational study where de‐identified samples were collected from a total of 6445 participants with (*n* = 2831, 44%) and without (normal controls, *n* = 3614, 56%) cancer. For the cancer cases, samples were only taken from participants who were newly diagnosed and treatment naïve. The age distribution of participants ranged from 20 to 80 years, with the majority (80%) falling between 30 and 80 years. Gender‐specific and age group‐specific distribution of the participants is summarized in Table S5, while Table S1 provides a more detailed profile of the individual participants. The cancer‐specific participant group covered a total of 30 cancers and Table S1 summarizes the overall distribution of these cases between the individual stages of the disease. As is evident here, over half (57%) of the participants in this group were in the early, or pre‐metastatic, stages (Stage‐I/II) of the cancer. Table 2 provides a list of the individual cancers, along with the gender‐ and stage‐wise distribution of participants for each of them.

### 3.2 Pre‐Processing of LC‐MS/MS Data

For analysis of the serum samples, we employed our previously described pipeline [41, 42, 43] wherein serum metabolomics was followed by analysis using a ML algorithm. The metabolome profile for each serum sample was generated by positive ion mode UPLC‐MS/MS, which resulted in over 20 000 spectral features (RT, *m/z* pairs). These features were subsequently resolved into known metabolites by employing the HMDB. The average number of known metabolites ranged from 2398 to 2801 for the individual cancers and normal controls (Figure S1), which then converged to a cumulative list of 8312 unique metabolites across all the groups. To next process this data, we modified our previously developed in‐house pipeline [43] consisting of normalization, gap filling, data transformation, prior to feature filtering and selection. We first performed a manual curation of the metabolite list to remove all metabolites of non‐human origin. Here, in addition to metabolites derived from either drug or plant products, we also removed those metabolites whose human origin could not be unambiguously ascertained. Finally, we employed a DF step wherein we excluded all metabolites that were present in less than 20% of the samples. The end‐product of this process was a matrix of 2709 features that represented samples from all the 6445 study participants (Section 2).

### 3.3 Development of a Machine Learning Algorithm to Distinguish Cancer Samples From Normal Controls

In our earlier study we had developed a supervised ML classification model, which we termed as the CDAI model, that could detect a total of 15 cancers in women with an average accuracy of around 99% [43]. However, since the goal of our present work was to cover a wider range of cancers in both females and males, we sought to further optimize the CDAI model by selecting a subset of samples from 21 of the 30 cancers for the purposes of training and testing. This resulted in a cancer‐specific group of 1445 samples. Additionally, 1812 normal control samples were also taken. The overall workflow employed for developing the CDAI model is depicted in Figure 1 whereas Table S2 describes the sample distribution employed, for both cancer and normal samples, between the training and test sets. The remainder of the samples (1386 cancer and 1802 normal control samples) were kept aside for subsequent validation of the CDAI model (Table S3).

A kernel based ML model SVM‐Rbf was used to train the model. Grid search was used to identify the optimal set of hyperparameters of the model, followed by a *k* (*k* = 20)‐fold cross validation. The results from *k*‐fold cross validation are shown in Figure 2, where we have evaluated the sensitivity of each fold. As is evident, the model was capable of maintaining the sensitivity across each fold in *k*‐fold cross validation score (Figure 2). To distinguish between cancer samples and normal control, the SVM‐Rbf classification function was applied to the training–testing dataset. By applying a cut‐off of zero for the resulting *y*‐scores generated for the individual samples in the train–test sample set, the sensitivity, specificity, and accuracy values obtained for the CDAI model were 98.4% (98.40%, 98.49%), 99.2% (99.20%, 99.25%) and 98.4% (98.84%, 98.89%) respectively. A detailed description of the CDAI model development steps is provided in the Section 2.

### 3.4 Validation of the CDAI Model

The developed model was validated using a distinct sample subset that had been separated at the beginning model building step. This validation set contained a total of 3188 samples of which 1386 were cancer samples from all the 30 cancer types and 1802 normal control samples. The sample distribution in the validation dataset is shown in the Table S3. This validation dataset was first pre‐processed as described in the Section 2, following which the CDAI model was applied to generate the scores for the samples. Using the cut‐off employed in the training–testing set we evaluated the sensitivity, specificity and accuracy obtained for the validation set. The CDAI model's train test accuracy was found to hold in the validation set as well, and we obtained a 97.5% (97.48%, 97.58%) sensitivity, 99.2% (99.18%, 99.23%) specificity, and 98.4% (98.46%, 98.52%) accuracy for the validation dataset. The score plot for samples in each of the 30 cancers, as well as those in the normal control group, generated by the SVM‐Rbf is shown in Figure 3A, whereas the individual values obtained for each sample in the validation set are given in Table S4. The confusion matrix that resulted for these 3188 validation samples is shown in Figure 3B. Further, we also evaluated the AUC‐ROC probability curve for the CDAI model using the samples from the validation dataset shown in Figure 3C. The model showed a high AUC‐ROC score of 0.99 for the validation dataset.

### 3.5 The CDAI Model Exhibits High Detection Sensitivity Across Cancer Types, Cancer Stages, and Subject Age Groups

**FIGURE 4:**
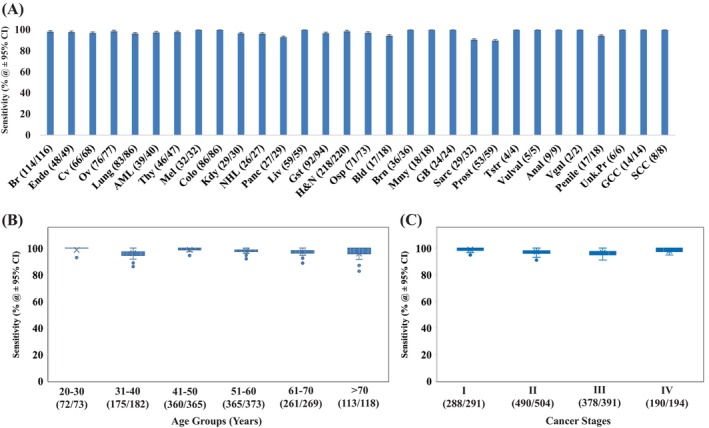
High fidelity performance of the CDAI is maintained across cancer types, subject age groups, and cancer stages. Panel (A) gives the sensitivity of cancer detection achieved by the CDAI for the validation subset of samples in each of the 30 cancer types tested. While the abbreviation for the cancers used here is the same as than in Figure 3A, the number of cancer‐positive samples identified, against the total number of samples tested, for each of the cancer types is included in parenthesis along the *X*‐axis. Panel (B) gives the CDAI cancer‐detection sensitivity obtained as a function of the age‐group distribution of the cancer patients while panel (C) shows a plot of detection sensitivity versus stage of the cancer. In both the latter panels the number of samples identified as cancer positive by the CDAI, versus the total number of validation set‐samples tested in each sub‐group is given in parenthesis along the *X*‐axis. For all panels, bars indicate 95% CI.

We next wanted to further dissect the results obtained for the validation set in Figure 3 to assess whether the cancer‐detection sensitivity showed any differences with respect to the cancer type being tested. Figure 4A shows a plot of the detection sensitivity, at 95% CI, obtained for each of the 30 cancers. While some cancer‐specific differences can be seen what is, nonetheless, noteworthy is the fact that all 30 cancers could be detected with a sensitivity that ranged from 90% to 100%, at a specificity of 99.2%. This included two cancers (prostate cancer and sarcoma) which were detected at a sensitivity of around 90% and an additional three (pancreatic, bladder, and penile cancers) where the sensitivity was between 90% to 95%. Detection sensitivity for the remaining 25 cancers exceeded 95% (Figure 4A). Thus, the CDAI algorithm does not exhibit any pronounced bias but, instead, performs equally well across all the cancers tested.

To determine whether there is any effect of age of the subject on test efficacy, we binned the cancer patients into six age groups and then estimated the sensitivity of the CDAI for each of these groups. As shown in Figure 4B, the sensitivity of > 96% was obtained across all age groups suggesting that the efficacy of the CDAI model will likely remain uniform across all ages in the adult population. It was also important for us to assess whether cancer‐detection capability of the CDAI algorithm fluctuated in any way depending on the stage of the disease. However, as shown in Figure 4C, this proved not to be the case and the model showed high sensitivity across all cancer stages. The stage wise sensitivity obtained was 98.9% for Stage‐I, 97.2% for Stage‐II, 96.6% for Stage‐III, and 97.9% for Stage‐IV cancers (Figure 4C). A particularly noteworthy aspect of these results is the high detection sensitivity obtained for the early‐stage (Stage‐I/II) cancers. These findings are significant in our view as they highlight that our method is uniquely capable of high‐fidelity detection of early‐stage cancers.

**FIGURE 5:**
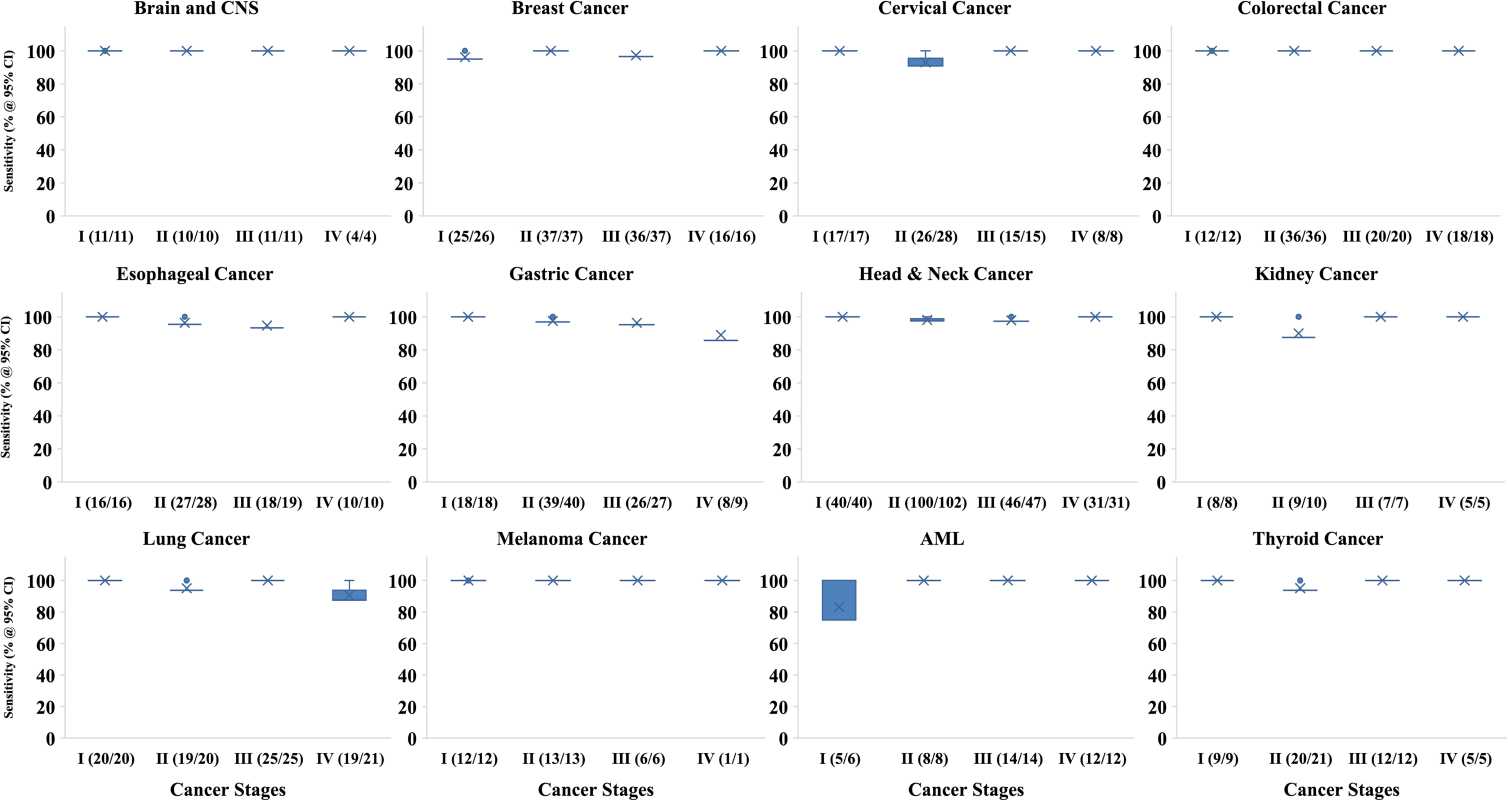
Stage‐specific CDAI test performance for representative cancers. Stage‐specific sensitivities obtained using the CDAI on the validation subset of 12 representative cancers are shown here. The number of samples identified as cancer positive, against the total number of samples tested at each stage of each of the cancers is also included in parenthesis and the bars indicate 95% CI.

Figure 5 profiles the cancer signal detection accuracy of our CDAI model, at 95% CI, for 12 representative cancers across the individual cancer stages. These results again highlight that the maximal cancer detection accuracy is already achieved by Stage‐I of the disease, and that this level is then largely maintained during subsequent progression of the disease through the remaining stages of II to IV (Figure 5). This trend was equally evident for all 12 cancers shown, implying that discernible changes in serum metabolome occur very early on during cancer development, which is then accurately captured by our CDAI algorithm.

### 3.6 Identification of Features Critical for Cancer Detection

The feature matrix generated using the VLM method uses the named metabolites from the HMDB database. Hence, the CDAI model can further provide valuable insights into cancer‐specific metabolic adaptations. Although ML models are fairly complex, feature ranking techniques can render relevant insights into classification patterns. To determine this, we paired SVM with Recursive Feature Elimination (RFE). Features were removed from the model iteratively and test sensitivity of the model developed from the remaining features was observed. Features giving rise to significant changes in the sensitivity were thus identified, and ranked on the basis of the extent of their influence on performance efficiency of the CDAI model. The top 100 named metabolites, along with their *m/z* values and observed retention times (RT), that resulted from this feature ranking exercise are listed in Table S5.

## 4 Discussion

As opposed to the more commonly adopted approaches of employing either ctDNA or CTCs for the development of MCED tests [44], we had chosen an alternate strategy of combining untargeted serum metabolomics with ML (ML)‐powered data analytics [41, 43]. In this connection, it is noteworthy that ML is fast emerging as an analytical solution for decoding metabolmics data, especially in the context of cancer detection [45]. Following our initial success at deploying this approach for concurrent detection of the four women‐specific cancers (breast, endometrial, cervical, and the ovarian cancers) [41, 42], we subsequently explored the versatility of this methodology by expanding its scope to cover a total of 15 cancers in women [43]. Here again we obtained excellent results where the overall detection accuracy achieved was around 99% [43]. As noted earlier [41, 43], a significant aspect of these findings was that the high detection accuracy was also retained for the early stage (i.e., Stage‐0/I/II) cancers. This aspect was particularly relevant given that early‐stage detection persists as a long‐standing challenge in the field of cancer diagnosis.

The present study underscores the adaptability of our method by demonstrating that it is multi‐cancer detection capabilities could be further expanded to cover a total of 30 cancers in both men and women. Importantly, this could be achieved without comprising the detection accuracy. Thus, all 30 cancers could be detected with a sensitivity that ranged from 90% to 100%, at a specificity of 99.2%. This included two cancers (prostate cancer and sarcoma) which were detected at a sensitivity of around 90% and an additional three (pancreatic, bladder, and penile cancers) where the sensitivity was between 90% to 95%. Detection sensitivity for the remaining 25 cancers ranged between 95% to 100%. It is pertinent to note here that the cancer types tested included both hematologic cancers (AML, NHL, multiple myeloma), as well as those that formed solid tumors. Our observation that age of the study participant did not confound the results is notable, as also is our earlier demonstration [43] that test accuracy was not in any way compromised by common co‐morbidities such as obesity, diabetes, heart disease, and hypertension among others.

Cancers, in general, are difficult to detect in the early stages because they are either largely asymptomatic or exhibit only vague and general symptoms that are not directly indicative of the disease. This problem is further exacerbated in the case of many solid cancers where the disease often starts deep in the tissue, which then further contributes to evasion of detection [46, 47, 48]. Consequently, majority of the cancers are diagnosed only at an advanced stage when prognosis is poor [49]. In the face of the continuing rise in cancer‐related deaths, early‐stage detection of the disease currently offers the only viable strategy for reducing morbidity and mortality due to this disease. The 5‐year survival rates are significantly higher in patients diagnosed with Stage‐I–II as opposed to those who are diagnosed at Stage‐III–IV [9]. Unfortunately, though, despite recent progress in the development of MCED tests, the goal of accurately diagnosing early‐stage cancer has remained elusive [27, 28, 29, 30, 31, 32, 33]. In this connection the combined findings from our earlier reports [41, 43] and the present study, that the approach of integrating untargeted serum metabolomics with data analysis using our CDAI algorithm enabled uniform detection of early stages (Stage‐0/I/II) of all the tested cancers with sensitivities of > 95%, is especially significant. They underscore the potential of our method for development as a multi‐cancer screening test which, because of its proficiency in detecting pre‐metastatic stages of the disease, could likely assist in reducing cancer mortality.

Even against the backdrop of the near universal difficulty of early‐stage cancer detection, there are some cancers that are particularly hard to discern. These are cancers that develop in tissues or organs that are deep inside the body and are, therefore, not readily accessible. Examples of these include pancreatic cancer, lung cancer, kidney cancer, ovarian cancer, sarcoma, brain cancer, liver cancer, and gallbladder cancer. Because of their comparative inaccessibility, tumours in these tissues or organs usually grow to a large size, and often metastasize, before they are detected. By this time, however, the 5‐year survival rates are substantially diminished [9]. Therefore, the results presented here indicating that even these difficult‐to‐detect cancers could be identified with high sensitivity in the early stages assumes special significance. Thus, although sample sizes were small, both Stages‐I and II of lung, kidney, brain and CNS, liver cancers, and sarcoma could be detected with 100% sensitivity. Detection sensitivities for Stage‐I and Stage‐II pancreatic cancer was 100% and 90% respectively, whereas that for ovarian cancer was 100% and 96% respectively. These results further support the prospective utility of our test for multi‐cancer screening. While our method employs LC‐MS/MS for generating the serum metabolome data, laser desorption/ionization mass spectrometry is also currently being explored as an approach for metabolome profiling towards cancer detection [50, 51]. It will be interesting to compare the two approaches in terms of generating the metabolite signatures required for accurate cancer detection.

While it is generally accepted that early diagnosis of cancer can save lives, mathematical modeling suggests that the objective of reducing cancer‐related mortality can be more effectively achieved through the use of MCED tests [52]. However, despite significant efforts in this latter direction, the goal of accurately detecting early‐stage cancers has persisted as an intractable problem. Nevertheless, results described in this report build on our previous findings to support that an interrogation of the serum metabolome with a ML algorithm, to capture disease‐related metabolite signatures, offers an effective way to detect all stages of cancers—including the early stages—with high fidelity. In this context the present study is especially noteworthy as it demonstrates the capability of our test to concurrently detect a total of 30 cancers in both adult females and males. Thus, the present test protocol possesses the ability to evolve into a multi‐cancer screening test that can be expected to contribute significantly towards reducing cancer‐related deaths. We do recognize, however, that further clinical studies are required to validate the utility of our test. To this end, a more extensive clinical trial has recently been initiated. Furthermore, we also realize that complementing cancer detection with identification of the tissue of origin (TOO) in the cancer‐positive cases would serve as an important value addition in terms of its clinical utility. Work on development of a TOO identification algorithm is also presently underway.

## Author Contributions

**Rajnish Nagarkar:** conceptualization, writing – review and editing, writing – original draft. **Mamillapalli Gopichand:** conceptualization, writing – review and editing, writing – original draft. **Suparna Kanti Pal:** resources. **Ankur Gupta:** software, formal analysis, methodology. **Najmuddin Md Saquib:** methodology, formal analysis, data curation. **Ahmad Ahmad:** software, formal analysis, methodology. **Ganga Sagar:** methodology. **Kanury V. S. Rao:** conceptualization, methodology, supervision, writing – review and editing, writing – original draft. **Zaved Siddiqui:** conceptualization, methodology, supervision, writing – review and editing, writing – original draft. **Imliwati Longkumer:** conceptualization, resources, data curation, writing – original draft, writing – review and editing.

## Ethics Statement

The study was approved by the Institutional Ethics Committee at North East Cancer Hospital and Research Institute (reference number IEC/04/06/2022) and Health Point Ethics Committee at Health Point Hospital (reference number HP/ EC/APVL/22‐23/011 dt. 19/may/2022), in accordance with the Declaration of Helsinki.

## Conflicts of Interest

A.G, G.S., Z.S., N.M.S., A.A., and K.V.S.R. are fulltime employees of PredOmix Technologies Private Limited. K.V.S.R. is a cofounder and owns stock in both PredOmix Technologies Private Limited and PredOmix Health Sciences Pte. Ltd. A.G., Z.S., and N.M.S. own stock in PredOmix Health Sciences Pte. Ltd. The work described in this report is included in the Patent Cooperation Filing. International application No. PCT/SG2024/050022. The other authors declare no conflicts of interest.

## Supporting information


Figure S1.
Figure S2.
Figure S3.

Table S1.

Table S2.
Table S3.

Table S4.

Table S5.

## Data Availability

The data that support the findings of this study are available from the corresponding author upon reasonable request.
