# Supplementary material for: Development of a Serum Metabolome‐Based Test for Early‐Stage Detection of Multiple Cancers
Source: Cancer Rep (Hoboken). 2024 Nov 19;7(11):e70042. doi: 10.1002/cnr2.70042 (PMC11574562; doi:10.1002/cnr2.70042)
Supplement: Supplementary file 1 — Figure S1. Figure S2. Figure S3. [file CNR2-7-e70042-s001.pptx]

## Slide 1
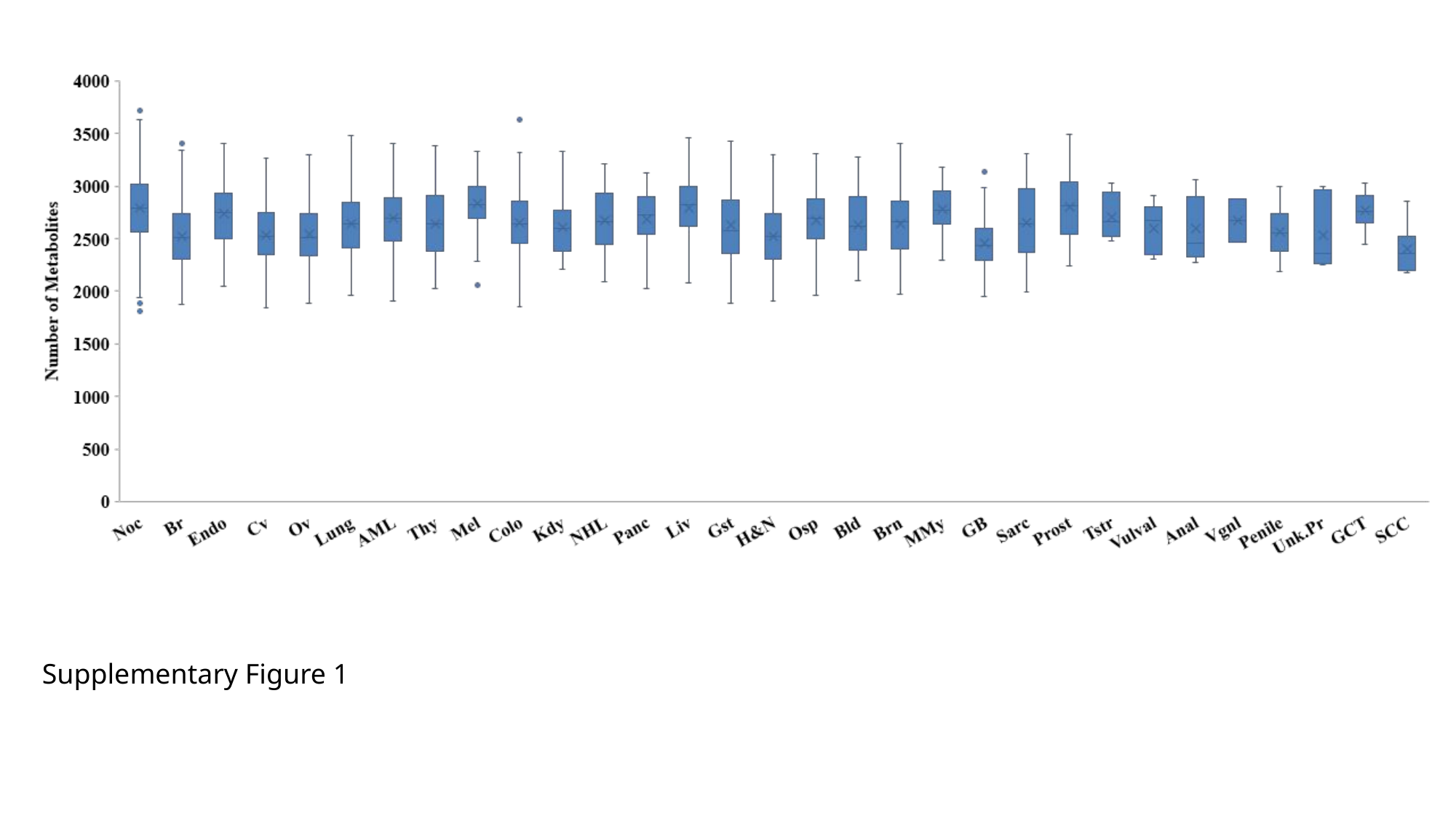

Supplementary Figure 1

## Slide 2
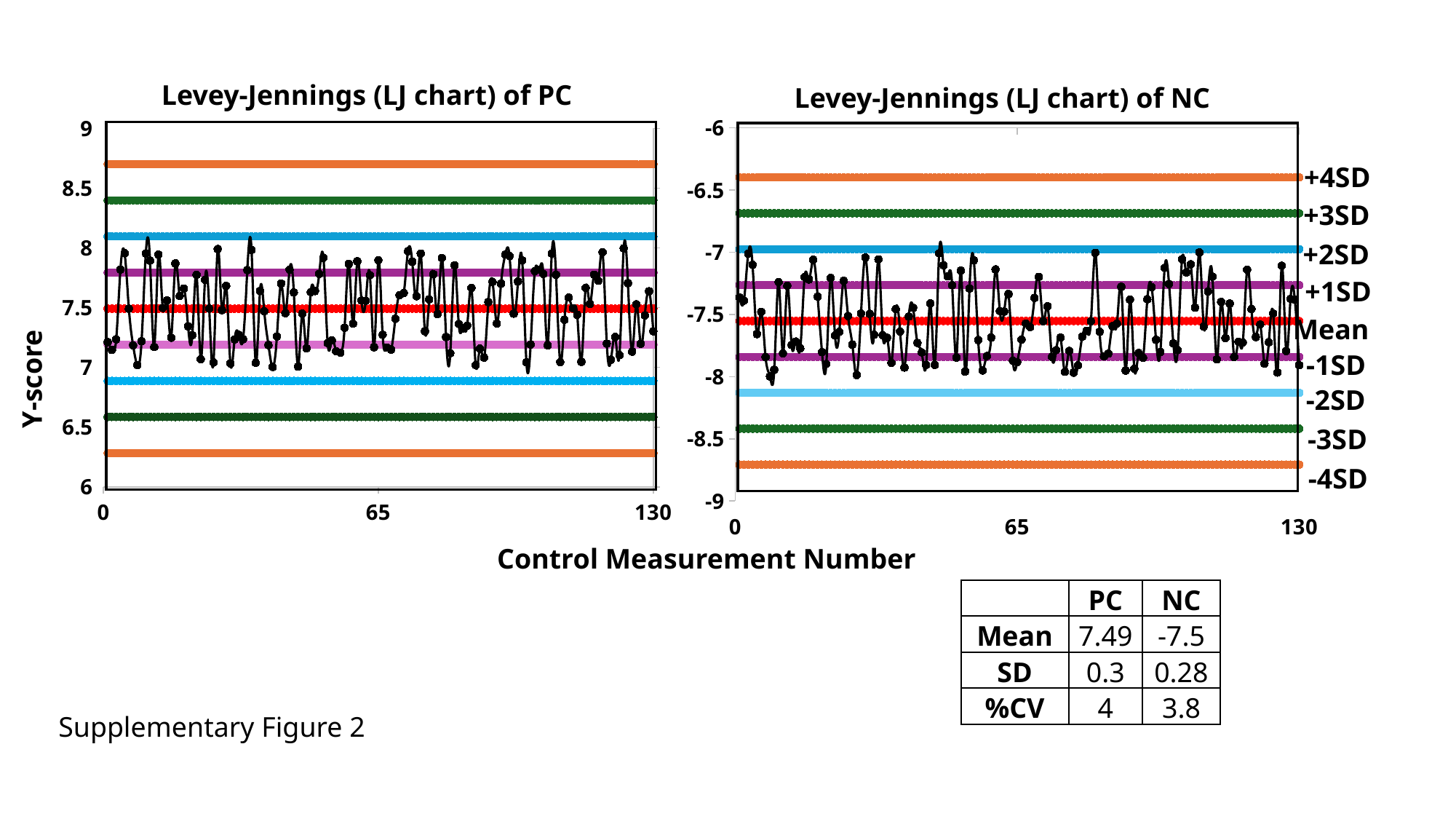

Levey-Jennings (LJ chart) of PC
Levey-Jennings (LJ chart) of NC
### Chart
| Category | Result | (+4SD) | (+3SD) | (+2SD) | (+1SD) | Mean | (-1SD) | (-2SD) | (-3SD) | (-4SD) |
|---|---|---|---|---|---|---|---|---|---|---|+4SD
+3SD
+2SD
+1SD
Mean
-1SD
-2SD
-3SD
-4SD
### Chart
| Category | Result | (+4SD) | (+3SD) | (+2SD) | (+1SD) | Mean | (-1SD) | (-2SD) | (-3SD) | (-4SD) |
|---|---|---|---|---|---|---|---|---|---|---|
Y-score
Control Measurement Number
| | PC | NC |
| --- | --- | --- |
| Mean | 7.49 | -7.5 |
| SD | 0.3 | 0.28 |
| %CV | 4 | 3.8 |
Supplementary Figure 2

## Slide 3
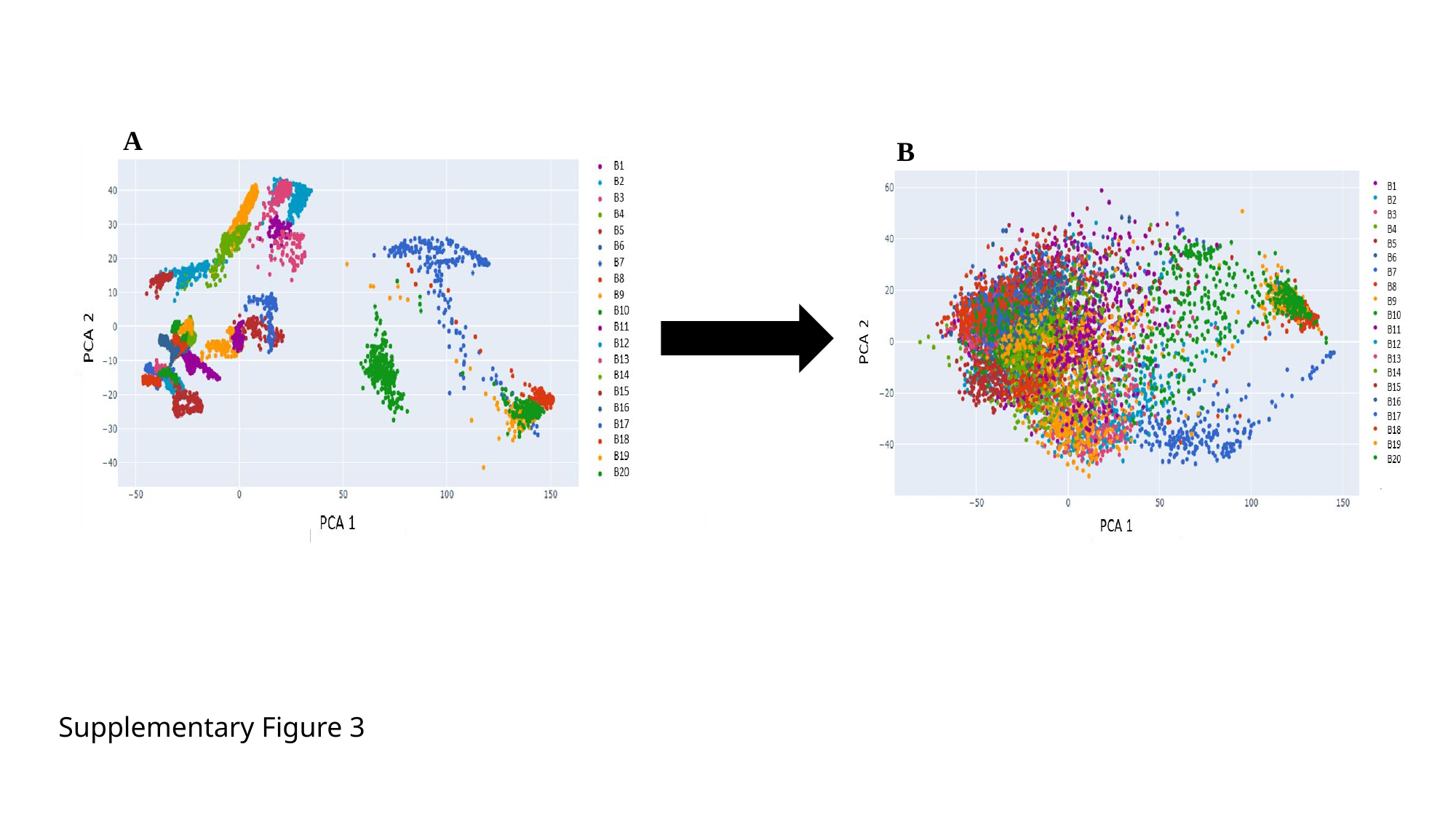

A
B
Supplementary Figure 3
