# Supplementary material for: Development of a Serum Metabolome‐Based Test for Early‐Stage Detection of Multiple Cancers
Source: Cancer Rep (Hoboken). 2024 Nov 19;7(11):e70042. doi: 10.1002/cnr2.70042 (PMC11574562; doi:10.1002/cnr2.70042)
Supplement: Supplementary file 3 — Table S2. Table S3. [file CNR2-7-e70042-s005.docx]

**Supplementary Table 2.** Sample set employed for training and testing the CDAI algorithm

| **No.** | **Cancer Type** | **Clinical Stage (*n*)**  **I II III IV** | **Total** |
| --- | --- | --- | --- |
| 1 | Breast | 10 96 40 - | 146 |
| 2 | Endometrial | 7 7 7 - | 21 |
| 3 | Cervical | 10 30 25 4 | 69 |
| 4 | Ovarian | 12 40 20 6 | 78 |
| 5 | Lung | 20 35 55 30 | 140 |
| 6 | ^*^AML | 4 7 15 20 | 46 |
| 7 | Thyroid | 7 25 20 - | 52 |
| 8 | Melanoma | 12 13 8 - | 33 |
| 9 | Colorectal | 8 45 70 - | 123 |
| 10 | Kidney | - 15 10 1 | 26 |
| 11 | ^*^NHL | 6 7 3 4 | 20 |
| 12 | Pancreatic | 1 15 10 4 | 30 |
| 13 | Liver & Bile | 16 16 16 6 | 54 |
| 14 | Gastric | 20 50 30 - | 100 |
| 15 | Head & Neck | 40 160 100 30 | 330 |
| 16 | Oesophageal | 10 33 23 4 | 70 |
| 17 | Bladder | - 6 8 - | 14 |
| 18 | Brain & CNS | - 3 3 - | 6 |
| 19 | Multiple Myeloma |  | NIL |
| 20 | Gall bladder | - 7 4 - | 11 |
| 21 | Sarcoma | - 3 10 - | 13 |
| 22 | Prostate | 7 20 30 6 | 63 |
|  |  | **Total** | **1445** |

**Supplementary Table 3.** Sample set employed for validating the CDAI algorithm

| **No.** | **Cancer Type** | **Clinical Stage (*n*)**  **I II III IV** | **Total** |
| --- | --- | --- | --- |
| 1 | Breast | 26 37 37 16 | 116 |
| 2 | Endometrial | 13 19 12 5 | 49 |
| 3 | Cervical | 17 28 15 8 | 68 |
| 4 | Ovarian | 16 24 28 9 | 77 |
| 5 | Lung | 20 20 25 21 | 86 |
| 6 | ^*^AML | 6 8 14 12 | 40 |
| 7 | Thyroid | 9 21 12 5 | 47 |
| 8 | Melanoma | 12 13 6 1 | 32 |
| 9 | Colorectal | 12 36 20 18 | 86 |
| 10 | Kidney | 8 10 7 5 | 30 |
| 11 | ^*^NHL | 7 8 6 6 | 27 |
| 12 | Pancreatic | 8 10 7 4 | 29 |
| 13 | Liver & Bile | 20 17 16 6 | 59 |
| 14 | Gastric | 18 40 27 9 | 94 |
| 15 | Head & Neck | 40 102 47 31 | 220 |
| 16 | Oesophageal | 16 28 19 10 | 73 |
| 17 | Bladder | 4 6 6 2 | 18 |
| 18 | Brain & CNS | 11 10 11 4 | 36 |
| 19 | Multiple Myeloma | 0 7 7 4 | 18 |
| 20 | Gall bladder | 3 11 9 1 | 24 |
| 21 | Sarcoma | 6 7 13 6 | 32 |
| 22 | Prostate | 10 17 25 7 | 59 |
| 23 | Testicular | 0 2 2 0 | 4 |
| 24 | Vulvar | 0 1 2 2 | 5 |
| 25 | Anal | 2 3 3 1 | 9 |
| 26 | Vaginal | 0 0 2 0 | 2 |
| 27 | Penile | 4 7 7 0 | 18 |
| 28 | Unknown primary origin | - - - - | 6 |
| 29 | Germ cell tumour | 0 10 3 1 | 14 |
| 30 | Squamous cell carcinoma | 3 1 3 1 | 8 |
|  |  | **291 503 391 195** | **1386** |
